# Preparation of Advanced Multi-Porous Carbon Nanofibers for High-Performance Capacitive Electrodes in Supercapacitors

**DOI:** 10.3390/polym15010213

**Published:** 2022-12-31

**Authors:** Donghui Zhao, Hui Wang, Yu Bai, Hao Yang, Hongfang Song, Baohua Li

**Affiliations:** 1 Shenzhen XFH Science and Technology Co., Ltd., Shenzhen 518071, China; 2 Shenzhen Key Laboratory of Power Battery Safety and Shenzhen Geim Graphene Center, Tsinghua Shenzhen International Graduate School (SIGS), Shenzhen 518055, China; 3 Shanghai XFH Science and Technology Development Co., Ltd., Building A7, Shanghai Future Office Park, Hutai Road, Shanghai 200949, China; 4 Key Laboratory for Green Chemical Technology of Ministry of Education, Haihe Laboratory of Sustainable Chemical Transformations, School of Chemical Engineering and Technology, Tianjin University, Tianjin 300072, China

**Keywords:** electrospinning, phenolic resin, carbon nanofibers, supercapacitor

## Abstract

The booming demand for energy storage has driven the rapid development of energy storage devices such as supercapacitors, and the research on high-performance electrode materials, a key component of supercapacitors, has gained tremendous attention. In this research, phenolic resin-based multi-porous carbon nanofibers have been prepared by electrospinning, curing, carbonization and activation and then employed as advanced electrode materials in supercapacitors. We demonstrate that the material is nano-scale continuous fiber, and its surface has pore distribution of different sizes. It delivers a high specific capacitance of 242 F g^−1^ at a current density of 0.2 A g^−1^ and maintains 148 F g^−1^ even at a high current density of 20 A g^−1^. Moreover, it shows almost no capacitance decay at a current density of 2 A g^−1^ over 1000 cycles, demonstrating its great potential as high-performance electrodes in supercapacitors.

## 1. Introduction

Due to the overconsumption of fossil fuels and environmental pollution, there is an urgent need to develop new renewable clean energy sources, such as wind energy [1] and solar energy [2], which however are unstable and intermittent [3]. Thus, energy storage technologies, such as fuel cells [4], batteries [5] and supercapacitors (SCs), have been developed in recent years. Among them, SCs are novel energy storage devices that can realize ultrafast charge storage and power delivery. According to the charge storage mechanisms, SCs can be generally divided into two categories: electric double layer capacitors and Faradaic pseudocapacitors. Although pseudocapacitors have higher energy densities, their electrode structures are not so stable over repeated redox reaction at the electrode surface during charge/discharge [6]. The electrode materials are mainly cost-expensive transition metal compounds, which hinders their wide applications [7]. In contrast, electric double layer capacitors with porous carbons as the electrode materials, have stable charge-discharge cycle and low capacitance attenuation rate. Owing to their high-power density, low maintenance cost and safe operation [8], they have been widely used in digital [9], transportation [10] and aerospace applications [11].

Since carbonaceous electrode materials play a critical role in determining the electrochemical performance of SCs, it has attracted extensive research attention to develop advanced carbon materials towards high charge storage capacity, high-rate delivery capability and excellent cycling stability [12,13,14]. Up to date, now zero-dimensional [15], one-dimensional [16], two-dimensional [17] and three-dimensional [18] carbon materials have been developed. As a typical one-dimensional material, carbon nanofiber material has attracted intensive research attention because of its large specific surface area, one-way efficient electron transport and easy construction of nano-devices [19]. Up to date, a considerable number of reports [20,21,22,23,24] prove the feasibility of its application in SCs. Electrospinning is regarded as an effective method to prepare nanofibers because of its simplicity and ease of controlling experimental conditions. By controlling the parameters, such as ambient temperature and voltage in the spinning process, the structure and morphology of nanofibers can be controlled, thus obtaining materials with ideal properties [25]. At present, carbon nanofibers prepared by electrospinning are mainly made of organic polymer materials, such as phenolic resin [26], polyacrylonitrile [27], and polyvinyl alcohol [28]. However, water-system-electrospun porous carbon nanofibers used for SCs have not been studied up to now. 

In this work, multi-porous carbon nanofibers (MPCNFs) with high specific surface area were prepared by electrostatic spinning from phenolic resin, carbonized at different temperatures and activated with ammonia, and supercapacitors were constructed to explore their capacitive charge storage properties.

## 2. Experimental

### 2.1. Materials Synthesis

#### 2.1.1. Materials and Equipment

Thermosetting phenolic resin (molecular weight 2080) was purchased from Durez Corporation (USA), and absolute ethanol (AR) was purchased from Beijing Hyundai Oriental Fine Chemicals Co., Ltd (Beijing, China). The main equipment used during the experiment is a high voltage generator (Beijing High voltage Technology Company, Beijing, China), an injection pump (model LSP01, Baoding Lange constant current Pump Co., Ltd., Baoding, China) and a tubular atmosphere furnace (model OTL1200, Nanjing University Instrument Factory, Nanjing, China).

#### 2.1.2. Preparation of Phenolic Resin Fibers

A certain amount of thermosetting phenolic resin was dissolved in absolute ethanol with a concentration of 27 wt.% and stirred in a constant temperature water bath at 35 °C for 12 h to form a uniform precursor.

Electrospinning the precursor solution into polymer fibers by electrostatic spinning device under the conditions of 25 kV voltage, 24–25 cm receiving distance and 1 mL h^−1^ feeding speed. After drying for 24 h, the phenolic resin fibers were cured by gradual heating to 180 °C at room temperature in an air atmosphere for 2 h.

#### 2.1.3. Preparation of MPCNFs

The cured fibers were carbonized in one step, and the temperature was raised to 650 °C, 750 °C and 800 °C at the rate of 5 °C min^−1^ in a nitrogen atmosphere; then ammonia gas was introduced for activation and held for 1 h. After heat preservation, the MPCNFs were obtained by cooling to room temperature in a nitrogen atmosphere. 

### 2.2. Characterization

The scanning electron microscope (SEM, LEO-1530, Germany) is mainly used to observe the fiber micromorphology; the transmission electron microscope (TEM, JEOL-2010, Japan) is used to observe the micromorphology structure of the fiber; thermal properties of cured fibers under N_2_ atmosphere were measured by thermogravimetric analyzer (TGA/DSC 1, METTLER TOLEDO, Switzerland); the X-ray photoelectron energy spectrum of (XPS, ESCALAB 250Xi, USA) is used to characterize the chemical composition and atomic state of the sample surface, calibrated using C1s characteristic peak 284.5 eV. The specific surface area and pore structural properties of the samples were obtained by analyzing the nitrogen adsorption desorption test at 77 K, using the equipment, the company’s Belsorp-Max physical sorbent instrument from Bel, Japan. Samples were degassed at 195 °C by nitrogen for approximately 18 h before testing. The X-ray fluorescence spectroscopy was used for elemental analysis to obtain the elemental content of the sample, using the instrument of XRF-1800 from Shimadzu, Japan.

### 2.3. Electrochemical Measurements

Due to the self-supporting structure and good conductivity of phenolic resin-based multi-porous carbon nanofibers prepared by electrospinning, there is no need to add additional conductive agent and binder when preparing electrodes. The prepared electrode was cut into a 1 × 1 cm^2^ square, sandwiched between 1 × 1 cm^2^ and 1 × 2 cm^2^ pieces of foam nickel and pressed into electrode sheets under the pressure of 2 MPa. The electrode sheet prepared by this method was fully soaked in 6 M KOH solution for 12 h.

The saturated calomel electrode (SCE) as the reference electrode, the platinum sheet electrode as the opposite electrode and the suppressed MPCNFs as the working electrode constitute a three-electrode system for electrochemical testing in KOH solution with a concentration of 6 M. Cyclic voltammetry (CV) and electrochemical impedance spectrum (EIS) are tested using the VSP300 electrochemical workstation, and the galvanostatic charge/discharge test (GCD) is conducted at the Arbin-BT2000 workstation.

## 3. Results and Discussion

**Figure 1:**
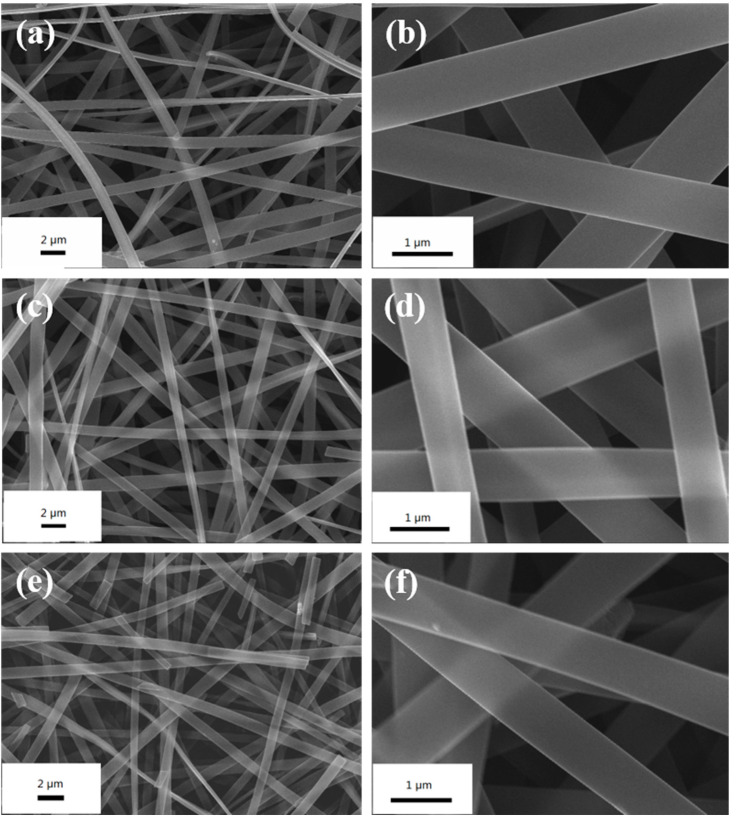
SEM images of (**a,b**) MPCNF-650, (**c,d**) MPCNF-750 and (**e,f**) MPCNF-800.

**Figure 2:**
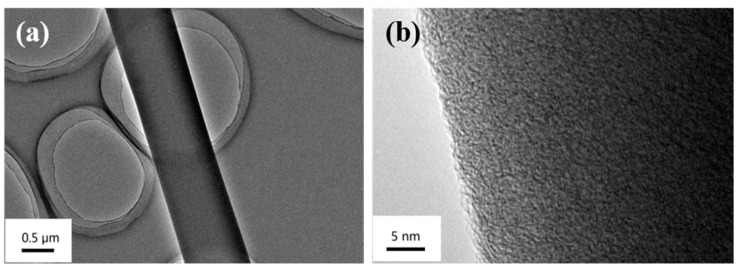
Low-magnification (**a**) and high -magnification (**b**)TEM images of MPCNF-750.

The phenolic resin fibers are heat treated at different high temperatures and the prepared products are designated as MPCNF-T (T is temperature). As shown in Figure 1, the MPCNF-T samples all showed nanoscale continuous fiber morphology with an average diameter in the range of 600–800 nm. With the increase of carbonization temperature, the fiber surface gradually changed from smooth to rough, and when the temperature reached 800 °C, continuous fibers began to break. Figure 2 shows the TEM spectrum of MPCNF-750. Under the condition of maintaining a relatively complete and continuous fiber morphology, the fiber surface is rough, and micropores with a pore diameter of about 0.5 nm are uniformly distributed.

**Figure 3:**
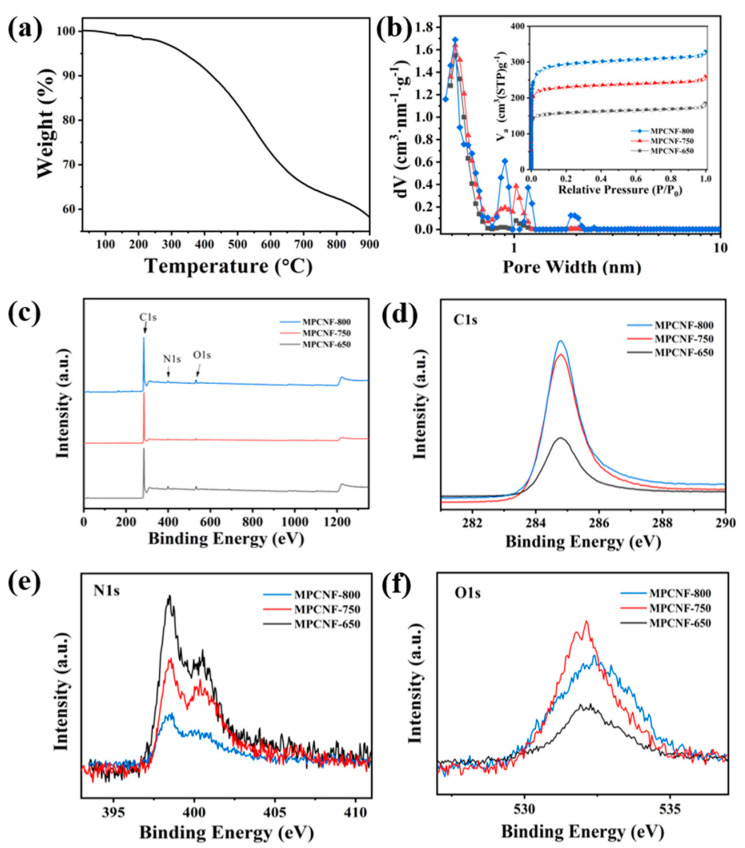
(**a**) Thermogravimetric curve of the cured polymer nanofibers. (**b**) Pore size distribution and isothermal adsorption-desorption curves (inset) of MPCNF−650, MPCNF−750 and MPCNF−800. (**c**) XPS spectrum of MPCNF−650, MPCNF−750 and MPCNF−800. High resolution spectra of (**d**) C1s, (**e**) N1s and (**f**) O1s for the different samples.

The thermogravimetric curve of the cured phenolic resin nanofibers is shown in Figure 3a. The cured nanofiber sample exhibits multistep weight loss due to loss of different species. The loss step from about 220 to 380 °C is ascribed to the loss of the –OH functional group of phenolic resin. Then the complete degradation of phenolic resin backbone and the elimination of hydrocarbonaceous residues lead to the steep loss step from around 380 °C, contributing to the formation of porous structure and surface chemical properties. 

The isothermal adsorption and desorption curves of MPCNF-T samples are shown in Figure 3b, all of which are I-shaped curves, indicating that the pore structure is mainly micropores. It can be seen from the figure that the nitrogen adsorption amount of the sample is positively correlated with the carbonization temperature, which is consistent with the morphological changes shown by SEM and TEM images (Figure 1 and Figure 2). The pore size distribution of the MPCNF-T samples in Figure 3b shows a graded pore structure, with the major pore size distribution of 0.5 nm small pores providing charge storage points and a certain amount of 1 nm pores acting as mass transfer channels and providing ion diffusion channels. When the carbonation temperature is increased from 650 °C to 750 °C, the number of pores around 1 nm increases, which is beneficial to improve the void utilization efficiency and to achieve high specific capacity and high-rate performance. However, when the carbonization temperature is too high, the number of large pores (especially those exceeding 1 nm in MPCNF-800 samples) increases, which accelerates the ion loss from the material and affects the electrical properties.

XPS measurement is used to study the surface chemical state of MPCNF-T, and the results are shown in Figure 3c. With the increase of carbonization temperature, the relative carbon content of the samples gradually increases, which shows that the characteristic peak intensity of carbon element in XPS gradually increases. The peak of N element also appears in the spectrum, which may be due to the residual N element when ammonia gas is added for activation. The pore parameters and relative contents of elements of the three samples are listed in Table 1. The surface area and pore volume of the samples gradually increased, and the elemental N content decreased as the heat treatment temperature increased, which is consistent with the results in Figure 3d–f. 

**Figure 4:**
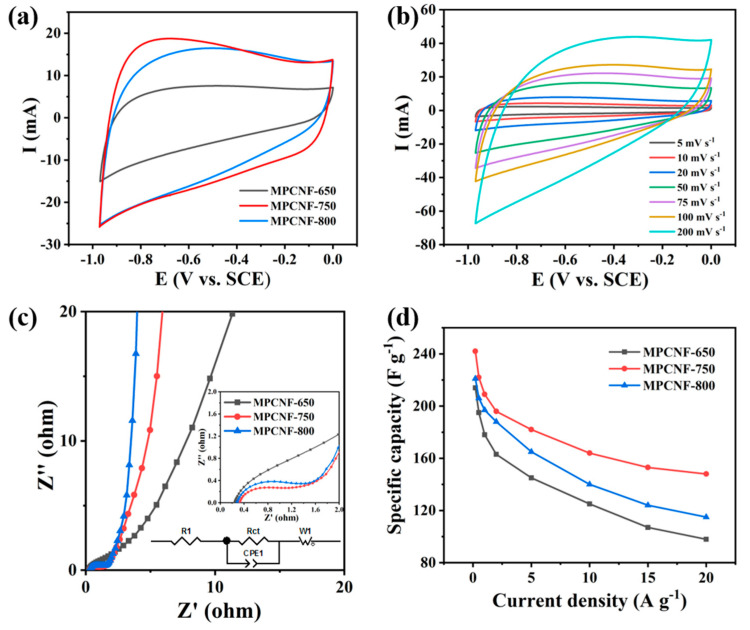
(**a**) CV curves of MPCNF−650, MPCNF−750 and MPCNF−800 materials at the scanning speed of 50 mV s^−1^. (**b**) CV curve of MPCNF−750 sample at different scanning speeds. (**c**) EIS spectrum of MPCNF−650, MPCNF−750 and MPCNF−800. (**d**) Comparison of specific volume values of MPCNF−650, MPCNF−750 and MPCNF−800.

The electrochemical performance of electrodes made of MPCNF-T was tested in a three-electrode system with voltage window of −1.0~0 V. Figure 4a shows the CV curves of three samples. The shape of CV curves is close to rectangle at the scanning speed of 50 mV s^−1^, which indicates that the material exhibits the characteristics of electric double layer capacitor. It can be seen intuitively from the figure that when the carbonization temperature of the material is 750 °C, the integral area of the CV curve is the largest, indicating that the specific capacitance obtained by MPCNF-750 is the largest. The stability of MPCNF-750 material was tested under the three-electrode system at different scanning speeds. As shown in Figure 4b, the CV curves of the material presented a stable rectangle in a large scanning speed range, until the CV curve of 200 mV s^−1^ was deformed, which indicated that MPCNF-750 material had good stability. To further explain the different electrochemical performances of the three materials, the EIS of the three materials were tested respectively, as shown in Figure 4c. In the Nyquist diagram, they consist of a depressed semicircle at high frequency, a 45° Warburg line at medium frequency and a straight slopping line at low frequency. The semicircle could be related to the charge transfer process at the electrode/electrolyte interface. According to the comprehensive EIS spectrum, MPCNF-750 material shows the lowest internal resistance, which benefits fast capacitive charge storage. Its schematic of the equivalent circuit of the supercapacitor is shown in Figure 4c. 

**Figure 5:**
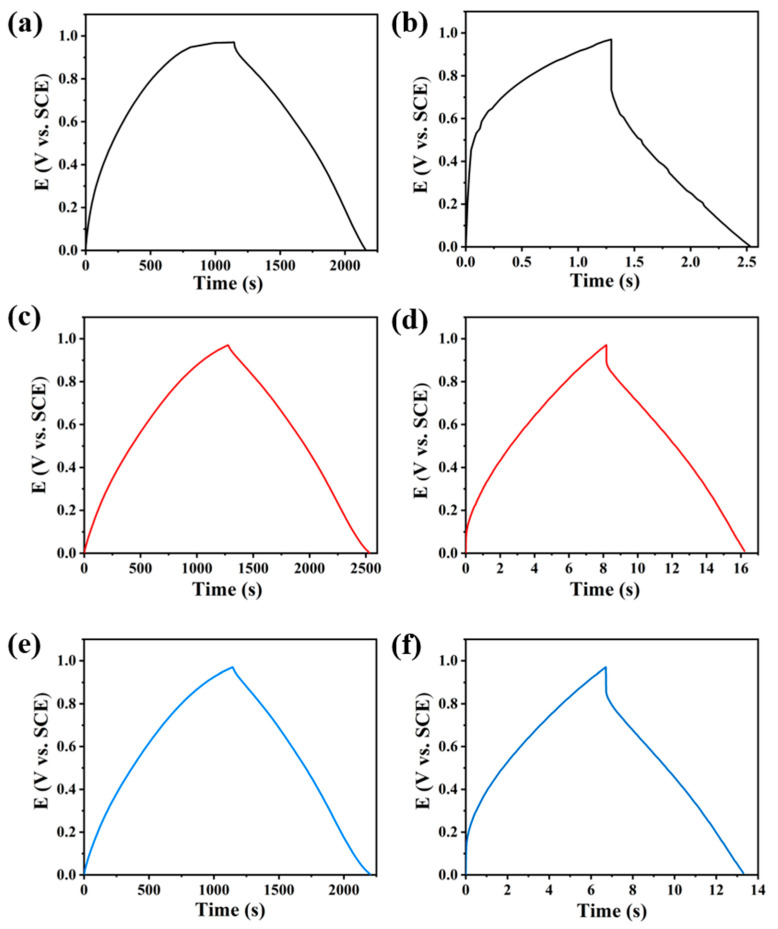
GCD curves of (**a,b**) MPCNF-650, (**c,d**) MPCNF-750 and (**e,f**) MPCNF-800.

The GCD properties of the three materials were tested at current densities of 0.2 A g^−1^ and 20 A g^−1^ respectively, and the results are shown in Figure 4d and Figure 5 (the specific capacitance of three samples is calculated according to the reported works [29,30]). The GCD curves of all three materials (see Figure 5) are similar to an isosceles triangle, and the charging and discharging time of MPCNF-750 material is the longest indicating its largest charge storage capacity, which is consistent with the results obtained by CV tests. Under high current density, the GCD curves of MPCNF-650 showed a certain degree of bending, which was caused by the disproportion of the material due to the high content of N elements. The properties of the material improved as the heat treatment temperature of the sample increased, i.e., the content of N elements decreased. The MPCNF-650 and MPCNF-800 materials have larger electrode polarization compared to MPCNF-750, and the discharging part of the GCD curve has a larger IR drop, resulting in a non-electrical double-layer capacitor characteristic, which leads to the degradation of the material performance. A comparison of the specific capacities of the three samples is shown in Figure 4d, in which the specific capacities of MPCNF-750 are higher than those of the other two samples in the current density range of 0.2–20 A g^−1^, and the specific capacity of 242 F g^−1^ is obtained at the current density of 0.2 A g^−1^. When the current density increases to 20 A g^−1^, the specific capacity decreases to 148 F g^−1^, and the capacity retention rate is 61.2%. By contrast, the capacity retention rates of MPCNF-650 and MPCNF-800 are only 45.8% and 52.0%, respectively. Such rate performance difference is consistent with the EIS testing results. Additionally, the energy density and power density of MPCNF-750 in the current density of 0.2 A g^−1^ are 33.6 Wh kg^−1^ and 295.6 W kg^−1^, respectively, according to the reported method of calculation [30,31,32]. 

**Figure 6:**
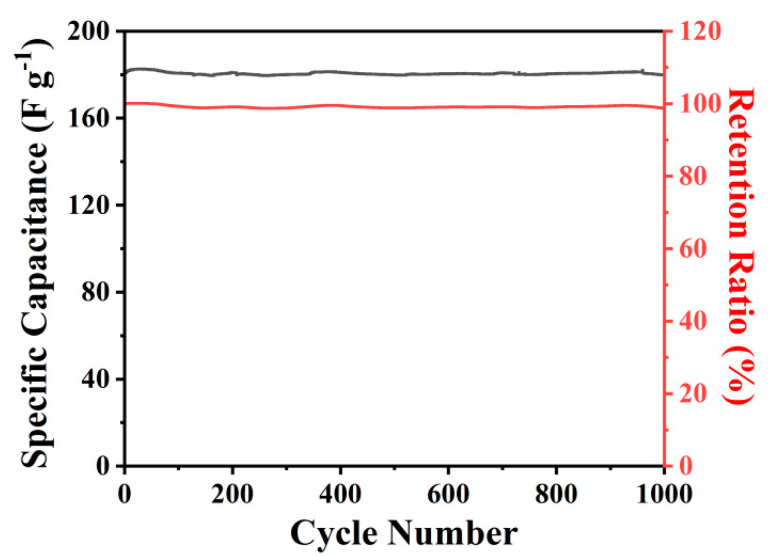
Cyclic performance of MPCNF−750.

To further investigate the cycling performance of MPCNF-750, the MPCNF-750 sample was selected as the working electrode and charged and discharged under a three-electrode system with a current density of 2 A g^−1^. The results in Figure 6 show that during the charge-discharge cycle, its reversible specific capacity was basically maintained at 180.0 F g^−1^ after the first 100 cycles of activation, showing relative cycle stability. After 1000 cycles, its reversible specific capacity was still obtained at 179.1 F g^−1^, with a capacity retention rate close to 100%. It can be seen that MPCNF-750 shows certain advantages in specific capacity and cycle stability, compared with the reported works [33,34,35,36] (Table 2).

## 4. Conclusions

Carbon nanofibers with high specific surface area and hierarchical porosity have been prepared by electrospinning phenolic resin, curing, carbonization and subsequent activation processes. As-obtained porous carbon nanofibers exhibit multi-peak pore size distributions, which benefits highly efficient utilization of the porous surface and fast ion transportation. Among all products prepared at different carbonization temperature, MPCNF-750 shows the best performance in terms of specific capacity, rate performance and cycling stability. It delivers a high specific capacitance of 242 F g^−1^ at a current density of 0.2 A g^−1^ and maintains 148 F g^−1^, even at a high current density of 20 A g^−1^ with little electrode polarization. Moreover, it shows almost no capacitance decay at a current density of 2 A g^−1^ over 1000 cycles. Therefore, as-prepared multi-porous carbon nanofibers hold great promise as high-performance electrodes for next-generation supercapacitors.

## Figures and Tables

**Table 1:** Porosity parameters and contents of C, N, O elements.

	TSSA ^a^ (m^2^ g^−1^)	MSSA ^b^ (m^2^ g^−1^)	TPV ^c^ (cm^3^ g^−1^)	MPV ^d^ (cm^3^ g^−1^)	C Content (at.%)	N Content (at.%)	O Content (at.%)
MPCNF-650	632	603	0.277	0.236	92.2	4.9	2.9
MPCNF-750	923	869	0.393	0.335	93.0	3.8	3.3
MPCNF-800	1165	1089	0.501	0.424	93.6	3.5	3.0

^a^ TSSA: Total specific surface area. ^b^ MSSA: Microporous specific surface area. ^c^ TPV: Total pore volume. ^d^ MPV: Microporous pore volume.

**Table 2:** Comparation of the electrochemical performance of MPCNF-750 with other reported works.

Materials	Current Density (A g^−1^)	Specific Capacity (F g^−1^)	Energy Density (Wh kg^−1^)	Power Density (W kg^−1^)	Cycle Retention (Cycle Number)	References
MPCNF-750	0.2	242	33.6	295.6	99.5% (1000)	This work
PANI/PEO	0.35	267	-	-	86.0% (1000)	[33]
Fe_3_O_4_/graphene	0.5	220	30.6	550.8	98.4% (3000)	[34]
Tetrahydroanthraquinone/reduced graphene oxide	0.2	234.8	-	-	95.6% (3000)	[35]
3D carbon nanotubes/poly (3, 4-ethylenedioxythiophene)	0.5	147	20.4	245.0	93.0% (200)	[36]
